# The IR-Homeostat Hypothesis: Intron Retention as an Evolutionarily Conserved Fine-Tuning Layer and a Reversible Blood Biomarker of Homeostatic Dysregulation in Mood Disorders

**DOI:** 10.3390/ijms27073119

**Published:** 2026-03-30

**Authors:** Norihiro Okada, Akiko Maruko, Kenshiro Oshima, Akinori Nishi, Yoshinori Kobayashi

**Affiliations:** 1 School of Pharmacy, Kitasato University, Tokyo 108-8641, Japan; 2 Tsumura Advanced Technology Research Laboratories, Research & Development Division, Tsumura & Co., Ibaraki 300-1192, Japan; 3 Oriental Medicine Research Center, School of Pharmacy, Kitasato University, Tokyo 108-8641, Japan

**Keywords:** intron retention, homeostasis, DGE, biological marker, stress, depression

## Abstract

Major depressive disorder (MDD) lacks reliable laboratory tests for diagnosis and treatment monitoring, underscoring the need for robust molecular readouts in blood. Beyond symptom-based classification, MDD can also be viewed as a condition involving impaired homeostatic regulation across stress-responsive, immune, metabolic, and neural systems. Consistent with this perspective, altered intron retention (IR) patterns have been observed in peripheral blood in depression-related and treatment-response contexts, supporting the translational relevance of this RNA-processing layer to mood disorders. A key observation underpinning this review is that IR can function as a reversible, intervention-responsive readout of physiological state. In a pre-symptomatic stress-like state in klotho mutant mice (a premature-aging model), widespread IR increases revert toward a healthy pattern upon treatment, suggesting that IR is embedded in a controllable homeostatic layer. Against the backdrop of limited cross-cohort transferability of differential gene expression (DGE) signatures, we propose that IR provides a mechanistically grounded biomarker layer because it reports regulated RNA processing states rather than context-fragile abundance endpoints. We operationalize IR as a post-transcriptional “throttle” on effective gene output, with increased IR/detained intron (DI) states acting as a reversible brake and decreased IR acting as an accelerator that increases translation-competent mRNA supply. Mechanistic exemplars across immune, metabolic, and neuronal systems (e.g., IFNG, OGT, MAT2A, neuronal activity-triggered intron excision, and intron detention-mediated stemness/differentiation switching in adult neural stem cells) show that defined inputs can switch IR/DI states to tune output kinetics. Integrating these findings, we propose an “Intron Retention Homeostat” (IR-Homeostat) model in which cells sense deviations from physiological set points and implement feedback control of gene output through switchable IR/DI regulation. This framework positions IR not only as a robust state readout for stratification, treatment response prediction, and pharmacodynamic profiling, but also as a tractable entry point to identify the molecular sensors and mediators that couple homeostatic signals to RNA processing control.

## 1. Introduction: Depression, Unmet Needs, and the Search for Blood Biomarkers

Clinical depression affects hundreds of millions of people worldwide and remains a leading cause of disability [1]. MDD is frequently accompanied by anxiety disorders and is a major contributor to suicide risk. Despite therapeutic advances, remission rates remain limited and decline with successive treatments, underscoring the need for objective biomarkers to monitor treatment outcomes and identify targetable pathways for intervention. Here, we consider intron retention (IR) as a regulatory RNA processing readout that can both report and potentially mediate stress adaptation.

**Figure 1:**
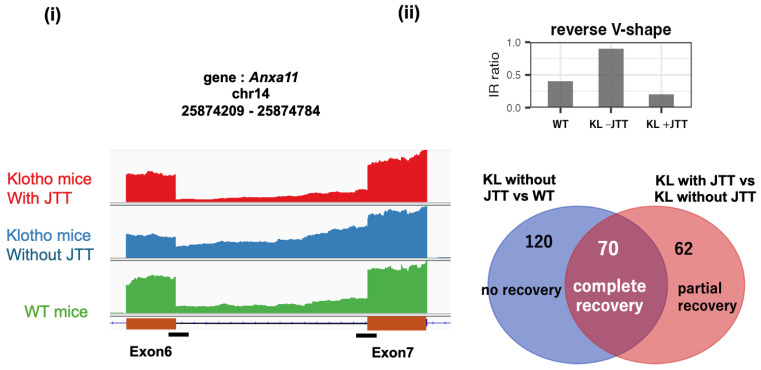
**Drug-responsive normalization of intron retention in a pre-symptomatic stress-like state.** (**i**) In klotho mutant mice (a premature-aging model), aging/stress-associated IR increases across loci (e.g., Anxa11), and a subset of IR events revert toward the healthy pattern upon juzentaihoto (JTT) treatment. Critical sites for evaluation of intron retention were shown by black bars. (**ii**) The Venn diagram summarizes 70 IR-affected genes whose retention levels recover toward the control pattern after treatment, suggesting coordinated regulation of IR across diverse transcripts (schematic adapted from Okada et al. [2]).

A motivating observation is that IR can behave as a reversible, intervention-responsive state marker rather than mere splicing noise. In a pre-symptomatic, starvation-like state in klotho mutant mice (a Klotho-deficient premature-aging model), retained introns accumulated across organs, and administration of the traditional multi-herbal medicine juzentaihoto (JTT) restored many IR events toward a healthy pattern (Figure 1) [2]. Importantly, the Venn diagram in Figure 1ii highlights that approximately 70 IR-affected genes show recovery toward the control pattern, implying that a shared upstream control mechanism can coordinately tune IR across diverse transcripts. Notably, JTT (and the related Kampo formula ninjinyoeito) also alleviates depressive-like behaviors and normalizes hippocampal neuroinflammatory and genome maintenance transcriptional programs in senescence-accelerated mouse prone 8 (SAMP8) mice, an accelerated-aging strain used as a depression-like model [3]. Independent work in SAMP8 mice further links depressive-like behavior to hippocampal neuroinflammation and shows that Kampo formulas can mitigate these emotional/inflammatory disturbances [4]. Consistent with this mood disorder-relevant context, hippocampal RNA sequencing (RNA-seq) in SAMP8 mice has also documented stress-associated IR accumulation in homeostasis “sensor” genes, with restoration toward the healthy state by Kampo medicines [5]. Together, these observations suggest that IR is embedded in a controllable homeostatic layer and set the stage for the IR-Homeostat model proposed later in this review.

Blood-based biomarker development has been motivated by feasibility and by growing evidence that peripheral inflammation correlates with depression incidence. Meta-analyses have reported elevated peripheral inflammatory markers—most robustly interleukin 6 (IL-6) and C-reactive protein (CRP)—in subsets of patients with major depression, although consistency varies across cytokines and study designs [6,7,8]. Preclinical aging models also support this inflammation–mood link. Specifically, SAMP8 mice exhibit depression-like behaviors accompanied by hippocampal neuroinflammation, and these changes can be buffered by Kampo formulas [4]. In clinical settings where inflammatory changes precede depressive symptoms, the hypothesis that inflammation contributes to depression has gained traction, supporting a framework in which blood is not merely a surrogate tissue but may capture upstream pathogenic drivers. Importantly, inflammatory signaling can regulate not only transcriptional abundance but also RNA processing states, making IR/DI a plausible interface where upstream homeostatic inputs are converted into output control.

Against this backdrop, we treat IR/DI as a mechanistically interpretable RNA processing layer for blood-based biomarker discovery. Building on the intervention-responsive IR normalization paradigm in aging/stress models (Figure 1) [2,9], we hypothesize that IR constitutes an early and reversible stress readout with utility for mood disorder biomarker development. We therefore organize this review around the idea that IR/DI acts as a post-transcriptional “throttle” on effective gene output, culminating in an IR-Homeostat model that links sensed deviations from physiological set points to switchable IR/DI regulation.

## 2. Why Differential Gene Expression Struggled as a Universal Blood Biomarker (Up to ~2016): Context Sensitivity and Cohort Instability

By the early-to-mid 2010s, many studies had attempted to derive blood-based diagnostic signatures for MDD using genome-wide differential expression analyses, predominantly with microarray platforms. A representative example is the report by Hori et al., [10] which noted highly variable findings across prior MDD transcriptome studies and combined discovery with candidate gene and pathway/network analyses in medication-free outpatients.

Whole-blood gene expression is an exceptionally sensitive, genome-wide, multi-gene state readout. Expression abundance can be shifted by infection and subclinical inflammation, sleep and circadian timing, diet, smoking and BMI, psychosocial stressors, and medication history. Technical and analytical factors further destabilize differential gene expression (DGE) rankings; batch effects can produce apparent group differences if not detected and corrected [11].

A particularly important issue is cellular composition heterogeneity. Whole-blood and peripheral blood mononuclear cell (PBMC) samples are mixtures of leukocyte subsets, whose proportions vary across individuals and can shift with stress and inflammation. Because many transcripts are cell type-enriched, mixture changes can masquerade as DGEs, reducing transferability across cohorts [12,13].

These features explain why DGE panels have struggled to become reliable, universal blood biomarkers. Specifically, the measurement target (steady-state mRNA abundance) is intrinsically state- and composition-sensitive, and it is further perturbed by technical variation. This motivates shifting biomarker emphasis from abundance endpoints to more regulated control processes.

## 3. The Bullmore Turning Point: Peripheral Inflammation as a Causal Driver Legitimatizes Blood Sampling

Bullmore’s “inflamed mind” framework proposed that, for a substantial subset of patients, peripheral inflammation can act upstream of depressive symptoms, providing a plausible causal route from systemic immune activation to altered mood and cognition [14]. If this framework is correct, blood becomes a legitimate compartment in which disease-relevant upstream drivers can be measured.

Crucially, this physiological framing does not exclude psychological causation. Negative life events, chronic adversity, and other psychosocial stressors can be conceptualized as upstream inputs that are translated into measurable physiological load through well-established pathways (e.g., neuroendocrine/autonomic signaling, sleep disruption, and downstream immune–metabolic changes). In this sense, blood-based markers capture the physiological implementation of psychosocial stress rather than the subjective experience itself. This provides a natural context to position RNA processing readouts, such as IR/DI, as candidate state variables: they may reflect how psychosocial and biological stressors jointly shift upstream regulatory states and how those states normalize during recovery.

Evidence that goes beyond simple cross-sectional association—such as temporal prediction and treatment-linked effects—strengthens this framework. Childhood IL-6/CRP levels predict later depression risk in population-based longitudinal data [15]. Immune activation during interferon-alpha (IFN-α) treatment is associated with subsequent depressive symptom trajectories [16]. Stratified intervention data suggest that baseline inflammatory status moderates antidepressant response to anti-inflammatory therapy in treatment-resistant depression (TRD) [17].

Although these findings strengthen the rationale for blood sampling, they do not resolve DGE instability. The translational task becomes identifying a mechanistically anchored readout that resists context dependence—an argument that motivates RNA processing regulation, particularly IR/DI.

## 4. The “Reproducibility Crisis” Does Not Imply Misconduct: A Framework That Explains DGE Instability

The reproducibility debate has largely framed irreproducibility as a structural and methodological challenge rather than a phenomenon attributable primarily to misconduct. Begley and Ellis argued that raising experimental standards is essential because influential preclinical results often fail to replicate under independent verification [18]. A *Nature* survey similarly reported that many researchers experience replication difficulties and perceive reproducibility as a major problem [19].

This framing is directly relevant to psychiatric biomarkers: failure to replicate can emerge from the interaction between a sensitive measurement target and heterogeneous human biology. Blood transcriptomics is intrinsically vulnerable because expression abundance is reactive to multiple state variables and strongly influenced by leukocyte composition. In addition, it is susceptible to technical variation (e.g., batch effects) [11,12,13].

A more promising strategy is to focus on molecular layers that represent regulated control processes rather than final abundance endpoints. This motivates emphasizing IR/DI as a candidate biomarker layer.

## 5. Intron Retention as a Stress-Responsive RNA Processing “Throttle”: IncIR (Brake) and DecIR (Accelerator)

IR is a form of alternative splicing in which introns remain within transcripts that would otherwise be fully spliced. IR is increasingly recognized as a conserved and regulated mode of gene control [20,21,22,23,24]. In many contexts, retained introns modulate effective gene output by controlling whether translation-competent mRNAs are produced and exported: intron-containing isoforms can be held in the nucleus and/or selectively removed by RNA surveillance pathways, thereby limiting the pool of mature mRNA available for cytoplasmic translation [24].

We conceptualize IR as a throttle controlling the effective supply of mature, export-competent (fully spliced; nuclear export competent) mRNA. Here, IncIR and DecIR denote the direction of IR change relative to the matched baseline (homeostatic) state. IncIR indicates a stress-associated increase in intron-containing precursor/IR transcripts (higher IR), whereas DecIR indicates a stress-associated decrease (lower IR). In this framework, IncIR functions as a brake, whereas DecIR functions as an accelerator. This model aligns with the view that IR can regulate effective gene output via nuclear retention and/or selective turnover routes, and with observations that IR signatures can normalize during recovery [2,9,24]. Notably, in metabolite homeostasis circuits (e.g., O-GlcNAc–OGT and SAM–MAT2A), the IR/DI throttle is explicitly bidirectional and reversible: returning the metabolite signal toward its set point restores retained intron states toward baseline [25,26].

Detained introns (DIs) are an operationally defined, mechanistically prominent subset of intron retention events: introns retained in nuclear polyadenylated transcripts with substantial half-lives, enabling regulated “holding” of transcripts until appropriate signals trigger splicing and release [27]. Because DI status is defined by nuclear enrichment/retention, it is formally established using subcellular fractionation or comparable assays; most bulk blood RNA-seq datasets (including those discussed here) do not separate nuclear and cytoplasmic compartments. Accordingly, throughout this review, we use “IR” in a practical, DI-like sense to denote regulated retained intron states detectable in bulk polyadenylated RNA, and we use “IR/DI” to emphasize the shared switchable control logic highlighted in our prior work. Consistent with this view, DI splicing can change rapidly in response to signaling perturbation [27]. In neurons, activity-dependent signals can trigger rapid splicing/export/ribosome loading, releasing mature mRNAs within minutes of stimulation [28]. Together, DI biology and rapid IR switching support IR/DI as a switchable, reversible control layer.

## 6. Mechanistic Exemplars: Stimuli Switch IR/DI States to Control Output Kinetics and Homeostasis

Across systems, this IR/DI control logic is repurposed for distinct physiological needs, and the direction of the switch (DecIR-like release vs. IncIR-like detention) is itself informative. In immune effector control, cytokine synergy provides a particularly clean example. Here, IL-12 primes IFNG transcription yet leaves a substantial fraction of intron-containing transcripts, and IL-2 signaling through nuclear factor kappa B (NF-κB) acts as a required secondary signal (“permission”) that drives productive processing to mature mRNA, rapidly boosting IFNγ output (DecIR-like acceleration). In metabolic homeostasis, DI switching implements feedback control. Specifically, OGT intron detention buffers O-linked β-N-acetylglucosamine (O-GlcNAc) under high-load conditions (IncIR-like brake) and is relieved when output must be restored (DecIR-like release), whereas S-adenosylmethionine (SAM) depletion promotes intron removal to increase MAT2A output. In fast neural programs, neuronal activity-triggered intron excision releases pre-existing transcripts on a minute scale (e.g., within ~15 min), without waiting for new transcription (elongation). Together, these cases argue that IR/DI switching is not pathway-specific noise but a regulated mechanism that tunes output kinetics and prevents overshoot or conflict by gating translation-competent mRNA availability in response to defined inputs [25,26,28,29,30]. Representative mechanistic exemplars across systems are summarized in Table 1.

Collectively, these examples support the central translational claim of this review: IR/DI captures regulated switching at control nodes, which may generalize more robustly across cohorts than steady-state abundance endpoints that are highly sensitive to environment and cell composition (Section 2, Section 3 and Section 4).

Expanded exemplar (adult neurogenic niche). González-Iglesias et al. demonstrate that intron detention can operate as an on/off switch that prevents conflicts between antagonistic transcriptional programs in adult neural stem cells (NSCs). Specifically, transcripts from differentiation genes are detained (intron-retained, nuclear-enriched) in NSCs, whereas stemness-related transcripts are preferentially processed and exported. Upon differentiation cues, this balance switches, and N6-methyladenosine (m6A)-dependent release of intron detention enables coordinated nuclear export and rapid output activation [31]. Importantly, their genome-wide analysis shows that IR/DI patterns separate early differentiation time points more clearly than DGEs (Figure 6 in [31]), consistent with IR as an early-state readout. In Figure 7 of [31], they provide representative examples of two opposite DI/IR programs that map naturally onto our IncIR/DecIR framework. Here, Cluster 1 follows a DecIR-like pattern (detention in NSCs, splicing upon differentiation), exemplified by Chd5, Sox6, Atl1, and Camk2a, whereas Cluster 2 follows an IncIR-like pattern (productive processing in NSCs, increased detention upon differentiation), exemplified by Kat2a, Lgr5, Fancc, and Ptprv. These paired examples provide a particularly clear demonstration that IR/DI switching can implement homeostatic or fate decision control by gating the supply of translation-competent mRNA.

Together, the exemplars in Table 1 illustrate why IR/DI can function as a robust state readout. By modulating the availability of translation-competent mRNA, IR/DI provides a reversible RNA processing control layer that can reshape protein output kinetics without requiring large de novo transcriptional remodeling.

## 7. Practical Implications: A Layered Biomarker Strategy Centered on IR/DI

Why overlap matters. A practical way to judge whether a molecular signature is likely to generalize is to ask whether it reappears across independent cohorts. Because steady-state abundance is highly sensitive to sampling conditions and differences in blood cell composition (Section 2, Section 3 and Section 4), DGE lists often show limited gene-level overlap between studies. By contrast, if IR/DI captures a more upstream and regulated layer of stress adaptation, IR gene sets should recur more reproducibly across cohorts even when ancestry, clinical stage, or sampling context differ.

Empirical example. This expectation is supported by our cross-cohort comparison (Figure 8D in Okada et al. [9]). IR gene sets (IncIR + DecIR) from a Japanese cohort at an early, subthreshold depressive stage overlapped strongly with two independent MDD cohorts: a Chinese case–control study [32] and a European treatment-resistant depression cohort enriched for ketamine nonresponders [33]. Despite differences in ancestry and clinical stage, pairwise IR overlaps were enriched 2.5- to 3.2-fold over random expectation (*p* < 0.0001), and 15 IR genes were shared across all three datasets. In contrast, the corresponding DGE comparison (Figure 8E in Okada et al. [9]) yielded only two genes shared across all three studies, with fold-enrichment values close to or below 1 (0.6–0.8), consistent with near-random overlap.

**Figure 2:**
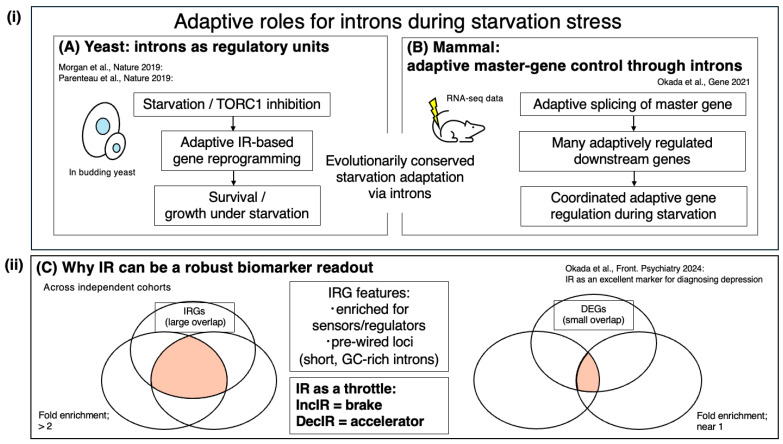
From ancestral starvation-responsive intron regulation to a conserved IR/DI homeostat: convergence across diverse stresses, evolution, and independent human cohorts. (**i**) Evolutionary continuity and expansion of starvation-responsive intron regulation. In yeast, nutrient starvation and/or TORC1 inhibition engages intron-based regulatory units that tune gene output to support growth control and survival, including both cis-acting starvation-responsive intron programs and trans-acting functions of excised intron RNAs [34,35]. In mammals, our motivating example—the klotho mouse model shown in Figure 1—exhibits a starvation-like metabolic signature (including elevated ketone bodies) together with widespread accumulation of retained/detained introns (IncIR), which can revert toward baseline during recovery and/or intervention [2]. Together, these observations support the idea that an ancestral starvation-responsive intron program was evolutionarily retained and expanded into a broader IR/DI-based homeostatic control layer in higher animals. (**ii**) Translational implication: distinct stress contexts, including inflammatory stress, can converge on partially shared IR control loci, promoting cross-cohort reproducibility of IR readouts. Conceptual Venn diagrams (adapted from [9]) illustrate that stress-responsive IR gene sets (IRGs) often exhibit substantial overlap across independent human cohorts (enrichment > 2), whereas DGE lists often show limited overlap (near-random enrichment ~1), reflecting the higher context sensitivity of abundance-based readouts [9]. IRG robustness is proposed to arise from pre-wired intronic control loci enriched in regulatory/sensor genes and characterized by short, GC-rich introns, where IR/DI acts as a post-transcriptional throttle on the supply of translation-competent mRNA (IncIR = brake; DecIR = accelerator). Venn diagrams are schematic and illustrate the principle rather than exact cohort sizes.

Taken together, these features place IR/DI readouts in a more favorable position than DGEs for subtype stratification and longitudinal monitoring within individuals. IR/DI reports upstream, reversible RNA-processing “throttle” states, whereas DGE primarily summarizes downstream abundance outputs that are more vulnerable to local sampling context and cell-mixture effects. This intuition is summarized schematically in Figure 2ii.

Why might this be the case? A plausible explanation is that IR often operates upstream of many downstream transcriptional outputs. In our previous analysis, genes recurrently showing IR were frequently annotated in the literature as sensors, regulators, or other control-node genes [9]. Such genes are well positioned to influence broad downstream programs even when their own effective output changes only modestly. In this framework, the key regulatory step is not necessarily a large change in total transcript abundance, but whether introns in nuclear pre-mRNAs are removed or retained, thereby controlling the supply of export- and translation-competent mRNA. As discussed in the mechanistic exemplars in Table 1, this principle is already established at specific loci. The broader implication is that, during homeostatic disruption, the same logic may be deployed across many regulatory genes, producing coordinated but potentially reversible shifts in effective gene output.

One way to conceptualize this hierarchy is to extend Bullmore’s military analogy of immunity, in which macrophages are stationed throughout tissues like “border guards or centurions” and lymphocytes function more like “generals” [14] (pp. 28, 34). In this framing, DGE readouts resemble rank-and-file soldiers: numerous downstream effectors whose abundance fluctuates with local “battlefield” conditions. By contrast, IR/DI can be viewed as exposing a higher-order command layer—effectively a “commander-in-chief” tier—in which pre-wired regulatory loci gate the supply of translation-competent mRNA [9]. If this interpretation is correct, IR gene sets should show stronger convergence across independent cohorts than DGE lists, because they report upstream control decisions rather than context-sensitive downstream abundance outputs.

Although blood cell composition can influence bulk transcriptomic outputs, the strong cross-cohort reproducibility of IR gene sets across ancestries and across PBMC versus whole blood suggests that the IR layer emphasized here is relatively robust to such variation. This does not mean that composition is irrelevant; rather, it indicates that a substantial core IR signal can persist across these sources of heterogeneity. In future clinical studies, composition-aware checks (e.g., CBC/differential counts or deconvolution) should therefore be viewed as tools for refining interpretation rather than as evidence against the utility of the IR layer itself.

IR should also be viewed as an independent—but complementary—biomarker layer relative to existing blood markers in depression. Established blood biomarkers include peripheral inflammatory markers such as CRP and IL-6, which index inflammatory load in subsets of patients [6,7,8]. These markers are clinically practical and useful for identifying immune-activated subtypes, but they do not report upstream RNA-processing states. We therefore position IR/DI as a complementary and partly independent biomarker layer: inflammatory markers quantify immune load, DGE summarizes downstream abundance outputs, and IR/DI reports regulated RNA-processing throttle states that are reversible and show stronger cross-cohort reproducibility than DGE lists [9,32,33].

In practice, the most effective strategy is likely to be layered rather than single-metric. For example, combining CRP/IL-6 with IR/DI state signatures may help distinguish high-load immune activation states from other stress programs and may improve longitudinal monitoring of treatment engagement and recovery. DGE panels can still provide pathway-specific effector information when composition and context are appropriately controlled [11,12,13]. Table 2 summarizes the complementary roles, strengths, and key confounds of IR/DI, DGEs, and inflammatory markers. A concrete illustration of this layered, upstream-oriented framework is provided by the ketamine cohort discussed in the next section.

In practice, an IR-centered workflow involves the following: (i) defining biological stress axes (e.g., inflammation and metabolic/oxidative stress); (ii) quantifying IR/DI throttle states at sentinel loci; (iii) enforcing robustness by requiring cross-cohort overlap and within-individual longitudinal normalization; (iv) connecting loci to mechanism via exemplars; and (v) translating the minimal set into targeted assays suitable for clinical use [2,6,7,8,9,11,12,13,25,26,27,28,29,30,36,37].

## 8. Ketamine as a Test Case for Upstream IR Robustness: Responder/Nonresponder Stratification and Pharmacodynamic Profiling

The ketamine cohort provides a concrete test case for the framework developed in Section 7. If IR/DI captures an upstream regulatory layer that is more stable and generalizable than downstream abundance outputs, then it should retain interpretable biological structure even within a clinically heterogeneous treatment setting. In this sense, the ketamine dataset is informative not only as a pharmacological perturbation study, but also as a practical demonstration that IR can reveal reproducible state information that is less obscured by downstream context dependence than DGE alone.

Cathomas et al. [33] generated whole-blood RNA-seq profiles in patients with treatment-resistant depression (TRD) before and after ketamine, thereby enabling interrogation of response heterogeneity. Building on these data, our IR-based reanalysis indicated that nonresponders were characterized by an elevated viral infection/innate immune activation state that emerged prominently through an IR-centric pathway structure [38]. This supports reframing nonresponse not as constitutional resistance, but as a subgroup with an immune activation burden that may prevent clinical response thresholds from being reached within the response window [38].

Importantly, this example also illustrates the practical advantage of an upstream IR/DI readout. Rather than merely cataloguing downstream expression differences at the “battlefield” level, IR highlighted a more coherent regulatory state that plausibly constrains treatment response. In other words, the ketamine cohort supports the view that IR can expose baseline response-limiting biology more robustly than DGE lists that are more vulnerable to local context, composition, and downstream effector variability.

IR can also function as a pharmacodynamic readout. Subsets of IR loci showed restoration toward healthy IR states after ketamine treatment regardless of responder/nonresponder classification, suggesting that IR can decompose drug-induced molecular normalization from clinical outcome divergence [38]. Thus, IR can simultaneously report the current state, baseline response-limiting biology, and the direction and magnitude of drug-induced normalization [33,38]. Together, these observations strengthen the argument that IR/DI is not merely another blood signature, but a relatively robust upstream biomarker layer that can support both stratification and longitudinal treatment monitoring.

## 9. Evolutionary Perspective: Introns as Units Regulating Homeostasis

The concept of homeostasis has been foundational in physiology for more than a century; however, its molecular implementation remains surprisingly under-specified in most discussions of gene regulation. In many contexts, we describe “homeostatic maintenance” as if it were an inherent property of cells, but it remains unclear whether a general molecular layer exists that actively senses deviations from optimal set points and restores balance through a defined regulatory circuit. Here, we propose that intron retention/detained introns (IR/DIs) may constitute such a mechanism: a switchable RNA-processing layer that converts homeostatic inputs into controlled changes in the supply of translation-competent mRNA, thereby buffering or accelerating protein output to stabilize cellular state.

**Figure 3:**
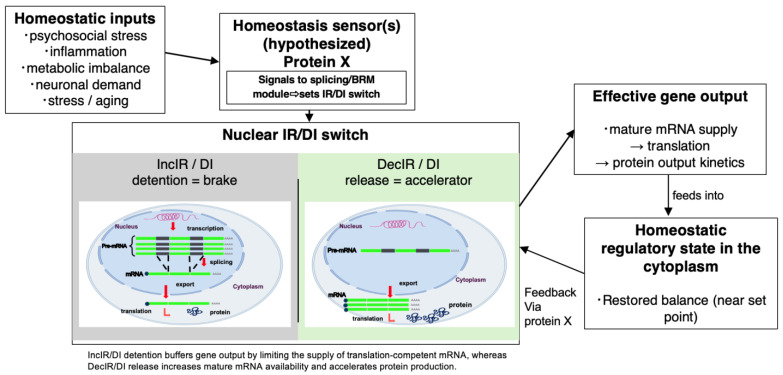
Proposed IR-Homeostat model: intron retention/detention as a switchable layer of homeostatic control. A conceptual schematic of how deviations from physiological set points are translated into coordinated IR/DI programs that regulate the effective supply of export- and translation-competent mRNA. A hypothetical homeostasis-sensing regulator (X) monitors inflammatory load, nutrient imbalance, or time-critical demand and, upon imbalance, translocates to the nucleus to bias RNA-processing decisions through the splicing machinery. Productive splicing (IR resolution) of pre-mRNAs encoding limiting proteins accelerates mature mRNA supply, whereas intron detention/retention in pre-mRNAs whose output should be constrained keeps transcripts nuclear and throttles the cytoplasmic supply of translation-competent mRNA. In this framework, X is presented as a conceptual shuttling RNA-binding regulator, and the figure illustrates the proposed logic of coordinated IR/DI control rather than an identified single molecule.

The IR-Homeostat hypothesis is consistent with the idea that introns can function as regulatory units across eukaryotes. In yeast, Parenteau et al. reported that introns mediate cellular responses to starvation, with systematic intron perturbations affecting fitness under nutrient limitation [34]. Starvation is among the most fundamental adaptive pressures, and our motivating mammalian example—the klotho mouse model depicted in Figure 1—shows a starvation-like metabolic signature, as evidenced by elevated ketone bodies, together with widespread IR accumulation that can be driven back toward a healthy pattern by intervention [2]. This parallel supports the view that intron-based buffering of gene output may be evolutionarily conserved. Morgan et al. further showed that excised linear introns can accumulate and regulate growth in yeast, demonstrating that intron sequences can retain biological activity even after splicing [35]. Together, these studies suggest functional diversification of intron-centered control. Specifically, introns can act in cis as switchable detention/retention elements that tune mRNA output during nutrient stress, whereas excised intron RNAs can act in trans to modulate growth programs, potentially converging on nutrient-sensing circuits such as TOR/mTOR. This evolutionary backdrop motivates the broader IR/DI framework summarized in Figure 3.

A useful way to integrate these observations is to regard starvation-responsive intron regulation as an ancestral prototype of a broader homeostatic RNA-processing logic. In higher animals, this logic appears to have been retained and repurposed beyond nutrient limitation, such that diverse deviations from physiological set points—including inflammatory, metabolic, and time-critical functional demands—can engage partially overlapping IR/DI control loci. Under this view, mammalian stress-responsive IR signatures are not isolated stress-specific curiosities, but diversified manifestations of a conserved intron-centered regulatory system that buffers gene output and helps restore physiological balance.

Dobzhansky’s dictum—“Nothing in biology makes sense except in the light of evolution” [39]—is therefore especially apt here. The starvation-responsive intron program in yeast [34], the starvation-like, ketone-associated IncIR accumulation in klotho mice (Figure 1) [2], and mechanistic IR/DI switching in mammalian immune, metabolic, and neuronal systems (Table 1) can be interpreted not as disconnected phenomena, but as evolutionarily related manifestations of the same general logic: intron-based control of effective gene output in response to deviations from homeostatic demand.

Importantly, IR/DI processes are not expected to involve all introns equally. In the IR-Homeostat framework, stress-responsive IncIR/DecIR events are expected to concentrate in a limited subset of cis-tunable “regulatory introns” (i.e., genetically specified control knobs) rather than reflecting random splicing noise. We return to the supporting evidence and factor-dependent intron subclasses in Section 10 [24,36,37,40].

Taken together, these findings suggest that intron-based regulation is not a mammal-specific curiosity, but an evolutionarily conserved and functionally diversified strategy for encoding environmental and homeostatic information. In mammals, retained/detained introns can be switched in response to defined inputs (Table 1), thereby tuning the availability of translation-competent mRNA and the kinetics of protein output; under this view, the IR signatures we observe in stress and recovery may represent the visible tip of a conserved intron-centered homeostatic control system. Viewed through this evolutionary lens, intron-based regulation provides a coherent backbone for the IR-Homeostat model and helps explain why partially shared IR loci may recur across distinct stress contexts and across independent human cohorts.

## 10. The IR-Homeostat Model: Intron Retention/Detention as a Switchable Layer of Homeostatic Control

To make the model structure explicit, we summarize the IR-Homeostat framework in four components (a–d).

- Core proposition. We propose the “Intron Retention Homeostat” (IR-Homeostat) model (Figure 3), in which cells sense deviations from physiological set points, such as inflammatory load, nutrient imbalance, or time-critical demand, and translate this information into switchable nuclear RNA processing states (IR/DI) that control the effective supply of export- and translation-competent mRNA. In this framework, IncIR/DI detention functions as a reversible brake that buffers output under stress, whereas DecIR-like release accelerates mature mRNA production when output must increase.
- Mechanistic logic. As illustrated by the exemplars in Table 1 (Section 6), homeostatic control can be implemented at the level of mRNA availability. Selected transcripts are held as intron-containing nuclear RNAs and can be rapidly converted into mature, export-competent mRNAs through regulated splicing and release. This gating layer can reshape protein-output kinetics without large de novo transcriptional remodeling, and it can prevent overshoot or conflicts between antagonistic programs by limiting the cytoplasmic mRNA pool until an appropriate context-confirming second input (“permission” signal) arrives (e.g., cytokine co-stimulation, metabolic sufficiency cues, or neuronal activity).
- Motivation and evidence. The model is motivated by the intervention-responsive normalization of widespread IR patterns in a pre-symptomatic, stress-like state (Figure 1) [2] and by mechanistic exemplars showing stimulus-dependent IR/DI switching across immune, metabolic, and neuronal systems (Table 1, including intron detention-mediated fate switching in the adult neurogenic niche [31]). Importantly, this control is not expected to involve all introns. IR/DI-prone loci often display distinctive cis-architecture (e.g., short and/or GC-rich introns with suboptimal splice signals), suggesting that “tunable” introns are genetically specified regulatory units rather than stochastic splicing failures. Consistent with this idea, specific classes of human short introns can be spliced in a factor-dependent manner (SPF45/RBM17, with SAP30BP as a cooperative component), demonstrating that alternative splice-factor logic exists for distinct intron subclasses [37,40].
- Testable predictions. Several concrete predictions follow from the IR-Homeostat framework. (i) If IR/DI represents a conserved homeostatic control layer, independent cohorts and distinct stressors should show partial convergence on a shared core of tunable IR loci, while retaining stressor-specific modules. Moreover, IncIR/DecIR patterns should track increasing stress load and tend to normalize during recovery. (ii) In time-resolved perturbations, IR/DI switching should occur earlier than—or partially decouple from—steady-state abundance changes, thereby better tracking output kinetics for selected pathways. (iii) The cis-architecture of tunable introns (length, GC content, weak splice signals, and decoy elements) should predict switchability, such that engineering these features alters detention/release dynamics. (iv) Interventions that restore physiology should drive IR signatures back toward a healthy pattern even when DGE endpoints remain heterogeneous. The next section outlines a practical roadmap to identify the mediators of these throttle decisions and to translate robust IR/DI loci into clinically deployable assays.

## 11. Clinical Translation

From a translational standpoint, the IR-Homeostat framework shifts the focus away from single time-point, cohort-dependent DGE signatures and toward within-individual longitudinal monitoring and subtype stratification using regulated IR/DI state switches. Discovery can be performed with genome-wide RNA-seq, whereas clinical deployment could rely on targeted assays that quantify a small panel of sentinel IR/DI events (e.g., junction-/intron-specific RT-qPCR, ddPCR, or targeted amplicon sequencing). Practical requirements include standardized pre-analytical procedures (blood collection, RNA stabilization, and quality control), consistent event-level normalization and reporting, and explicit consideration of blood cell composition effects. Together, these steps enable cost-effective pharmacodynamic monitoring in clinical cohorts. In addition to potential diagnostic utility (including comparisons with other psychiatric disorders), the IR/DI layer may be particularly well suited to patient monitoring and follow-up. Within-individual trajectories can provide pharmacodynamic readouts of treatment engagement, remission versus non-remission, relapse risk, and the risk of somatic comorbidities (e.g., cardiometabolic and inflammatory disease). Because homeostatic dysregulation and allostatic load are transdiagnostic, future studies should test IR/DI panels across disorders such as bipolar disorder, anxiety disorders, and post-traumatic stress disorder (PTSD), and extend evaluation to early cognitive decline (mild cognitive impairment; MCI), where sensitive, longitudinal biomarkers are needed to track progression and intervention effects.

## 12. Limitations and Future Directions: From Biomarker Phenomenology to Mechanistic Control

Several limitations and near-term priorities shape the path from descriptive blood signatures to a mechanistic, clinically usable IR/DI framework:

- IR calling remains sensitive to read depth, annotation, and thresholding. Because intronic signal is relatively sparse in typical poly(A) RNA-seq, sequencing depths that are adequate for DGE analysis can be underpowered for robust IR/DI detection. Splicing-oriented evaluations suggest performance becomes relatively stable beyond approximately 40–60 million reads per sample, whereas comprehensive splice event detection can require more than 100 million reads, a key caveat when re-using DGE-centric public datasets. Consensus standards for IR-aware QC, normalization, and longitudinal comparability are required [36,41,42,43].
- Bulk blood conflates immune subsets; composition effects remain relevant and must be addressed using sorting/single-cell approaches and composition-aware modeling (e.g., complete blood count with differential (CBC), cell #type deconvolution, or sensitivity analyses adjusting for estimated cell proportions) [12,13]. In depression, leukocyte and platelet indices such as the neutrophil-to-lymphocyte ratio (NLR), monocyte-to-lymphocyte ratio (MLR), platelet-to-lymphocyte ratio (PLR), and platelet count are frequently altered, providing clinically accessible proxies for immune-balance shifts and allostatic load [44,45,46,47]. Furthermore, platelets, although anucleate, show activation-dependent splicing of resident pre-mRNAs that can yield functionally relevant proteomic changes [48]. As well, these robust and upstream analyses of blood IR/DI signatures should be complemented with downstream proteomic and metabolomic analyses that express the phenotype, providing a highly detailed and integrated picture of variations that underlie patient status and treatment response.
  These parameters could be intertwined with observed IR/DI patterns and can be incorporated as covariates or stratification axes in future cohorts. Linking IR/DI switching to allostasis and set-point changes remains a key priority. Chronic stress and inflammation can reset physiological set points; we hypothesize that such set-point shifts will manifest as baseline shifts in the IR/DI “throttle” state, with effective interventions reversing these shifts. Longitudinal within-individual designs will be essential to quantify set-point drift, hysteresis, and recovery kinetics.
- Although mechanistic exemplars exist (Table 1), we still lack a mood disorder-specific map that connects clinically relevant inputs (e.g., inflammatory, metabolic, or stress-related cues) to the specific mediators and IR/DI “switch” loci they engage. Perturb-and-measure experiments and CRISPR-based genetic screens provide a practical roadmap to build this causal chain [25,26,27,28,29,30].
- Prospective cohorts with repeated measures are needed to test response prediction, relapse risk, and individual-level normalization [2,9]. Ketamine datasets illustrate that IR can stratify nonresponders by immune activation burden and capture drug-induced normalization, supporting evaluation of IR as both predictive and pharmacodynamic biomarker [33,38].
- Different stressors may recruit overlapping but non-identical IR modules; defining core versus context-specific IR programs is essential for generalization [9,36].
- Model systems that show reversible, drug-responsive IR shifts provide tractable tests for mechanisms. As shown in the klotho model (Figure 1) [2], IR accumulation in a pre-symptomatic, starvation-like state can be selectively normalized by juzentaihoto. Complementing this blood-centric framework, central nervous system (CNS)-focused datasets (e.g., hippocampal RNA-seq in depression-like SAMP8 mice) and Kampo interventions offer a tractable entry point to test IR/DI predictions in the brain [3].

## 13. Concluding Remarks

Across diverse biological systems, IR/DI behaves as a regulated, switchable RNA processing layer that gates the supply of translation-competent mRNA and thereby tunes output kinetics. In mood disorder research, this property offers a practical advantage. Specifically, IR-based signatures appear more robust to cohort and sampling variation than steady-state abundance endpoints, supporting cross-cohort generalization, subtype stratification, and longitudinal monitoring.

We propose the IR-Homeostat model as a unifying framework that links mechanistic exemplars of detention/release to clinically meaningful “state load” axes, such as inflammatory and metabolic stress. Testing this framework will require standardized IR quantification, cell type-resolved profiling, time-resolved perturbations, and genetic dissection of the regulators that couple signaling inputs to intron switch states.

If successful, an IR-centered strategy could provide both interpretable biomarkers and mechanistic entry points for intervention, enabling separation of molecular normalization from symptom-based thresholds and helping to map how homeostatic stress adaptation is implemented at the RNA processing level.

## Figures and Tables

**Table 1:** Mechanistic exemplars of stimulus-dependent IR/DI switching that gates effective gene output.

Input/Trigger	System	Target Gene/Locus	IR/DI Switch (Mechanism)	Output Consequence	Key Refs.
Cytokine synergy: IL-12 +/− IL-2	NK cells	IFNG	IL-12 primes IFNG transcripts with introns retained; IL-2 triggers NF-κB-dependent splicing state switching (largely independent of nascent transcription)	Mature IFNG mRNA and IFNγ protein output increase synergistically	[29]
O-GlcNAc homeostasis: high vs. low O-GlcNAc	Human cells	OGT intron 4 (DI)	High O-GlcNAc favors intron detention/nuclear retention; low O-GlcNAc favors rapid excision/export	Cytoplasmic OGT mRNA decreases (high)/increases (low)	[25]
Trans-acting factor control via decoy-exon logic (decoy exon; a regulatory exon-like element)	Human cells (CRISPR screen)	OGT decoy exon within DI	SFSWAP regulates OGT intron detention; depletion enhances productive splicing and alters decoy usage	Mechanistic lever for DI control; suggests broader DI/decoy programs	[30]
System-wide DI tuning by O-GlcNAc	Mammalian cells	Multiple DI loci	O-GlcNAc levels broadly modulate DI splicing	Global DI shifts with minimal changes in other alternative splicing modes	[25]
SAM depletion (methionine starvation)	Human cells	MAT2A retained intron	SAM depletion induces splicing changes that increase MAT2A expression (METTL16-linked)	Restores SAM synthesis capacity	[26]
Neuronal stimulation	Neurons	Multiple transcripts	Targeted IR/excision enables transcription-independent rapid release	Minute-scale transcript availability	[28]
Neural differentiation signal (adult neurogenic niche); m6A-dependent release	Adult neural stem cells (NSCs)	Representative genes (Figure 7 in [31]) Cluster 1 (DecIR-like): Chd5, Sox6, Atl1, Camk2a Cluster 2 (IncIR-like): Kat2a, Lgr5, Fancc, Ptprv	Two opposite DI/IR programs during NSC→differentiation. Cluster 1: detention in NSCs → splicing + export upon differentiation (DecIR-like release) Cluster 2: productive processing in NSCs → increased detention after differentiation (IncIR-like brake)	Fate-switch control by gating translation- competent mRNA availability; IR/DI separates early differentiation time points (Figure 6 in [31])	[31]

Representative examples across immune, metabolic, and neuronal systems illustrate how defined inputs (e.g., cytokine co-stimulation, O-GlcNAc or SAM availability, neuronal activity, and differentiation cues) trigger switch-like intron retention/detention (IR/DI) control at specific loci in key regulatory genes. In the IR-Homeostat framework, IncIR/DI detention acts as a reversible “brake”, whereas DecIR/DI release/excision acts as an “accelerator”, thereby modulating the supply of translation-competent mRNA and tuning protein output kinetics. Columns list the input/trigger, biological system, target gene/locus, IR/DI switching mechanism, functional output consequence, and key references. While all exemplars illustrate switchable IR/DI control, bidirectional reversibility is most explicitly demonstrated in metabolite homeostasis circuits such as O-GlcNAc–OGT and SAM–MAT2A [25,26].

**Table 2:** Complementary and partly independent roles of IR/DI, DGEs, and inflammatory markers as blood-based readouts in depression.

Readout (Layer)	Primary Signal	Typical Strengths	Key Limitations/Confounds	Added Value/ Complementarity	Key Refs
IR/DI (event-level RNA processing)	Upstream RNA processing state controlling the effective supply of export- and translation-competent mRNA through intron excision or detention/retention.	Reversible; pharmacodynamic state readout; stronger cross-cohort reproducibility than DGE lists; suitable for subtype stratification and within-individual longitudinal monitoring.	Requires RNA-based assay and event-level calling; sensitive to RNA quality, read depth, and pre-analytical handling; some events may be modulated by blood cell composition, although a core IR layer appears relatively robust across cohorts and across PBMC versus whole blood.	Adds an upstream regulatory “throttle-state” layer that is partly independent of inflammatory load markers and can track treatment engagement, recovery, and stress-state normalization; complements CRP/IL-6 (immune load) and DGEs (downstream outputs).	[9,32,33]
DGE signatures (abundance output)	Downstream steady-state mRNA abundance changes integrating multiple upstream regulatory steps.	Established workflows; intuitive pathway-level interpretation; useful for identifying effector programs when composition and context are well controlled.	Highly sensitive to sampling context, batch effects, and leukocyte composition; often shows limited gene-level reproducibility across cohorts.	Provides downstream effector readouts that can be interpreted in light of upstream IR/DI state shifts and inflammatory load.	[10,11,12,13]
Inflammatory markers (CRP, IL-6, etc.)	Peripheral inflammatory load at protein-level.	Inexpensive and standardized assays; clinically familiar; useful for identifying immune-activated subtypes and estimating allostatic burden.	Nonspecific; influenced by infection, obesity, medication, and comorbidities; limited resolution for upstream RNA-regulatory state.	Defines immune-load context and can be combined with IR/DI to distinguish immune-activated stress states, interpret nonresponse constraints, and improve longitudinal monitoring of recovery trajectories.	[6,7,8,14,15,16,17]

## Data Availability

The RNA-seq data analyzed shown in Figure 1 are publicly available in the DDBJ Sequence Read Archive (DRA) under BioProject PRJDB7898 (run accessions: DRR167982–DRR167990; DRR259222–DRR259248). These datasets were originally generated and reported in [2] (Okada et al., Gene 2021, 794:145752). No new sequencing data were generated in this study.

## Abbreviations

APEX2	engineered ascorbate peroxidase 2
CBC	complete blood count
CNS	central nervous system
CLK	CDC-like kinase
CRP	C-reactive protein
ddPCR	droplet digital PCR
DGE	differentially expressed gene(s)
DI	detained intron(s)
IFNγ	interferon gamma
IFNG	interferon gamma (gene)
IL-6	interleukin 6
IR	intron retention
JTT	juzentaihoto
m6A	N6-methyladenosine
MDD	major depressive disorder
METTL16	methyltransferase-like 16
MLR	monocyte-to-lymphocyte ratio
NMD	nonsense-mediated decay
NF-κB	nuclear factor kappa B
NK	natural killer
NLR	neutrophil-to-lymphocyte ratio
NSC	neural stem cell
O-GlcNAc	O-linked β-N-acetylglucosamine
OGA	O-GlcNAcase
OGT	O-GlcNAc transferase
PBMC	peripheral blood mononuclear cell(s)
PLR	platelet-to-lymphocyte ratio
PTSD	post-traumatic stress disorder
QC	quality control
RNA-FISH	RNA fluorescence in situ hybridization
RT-qPCR	reverse transcription quantitative PCR
SAM	S-adenosylmethionine
SAP30BP	SAP30 binding protein
SF3B1	splicing factor 3B subunit 1
SON	SON DNA/RNA-binding protein
SPF45/RBM17	RNA-binding motif protein 17
SRRM2	serine/arginine repetitive matrix 2
TNF	tumor necrosis factor
TRD	treatment-resistant depression
U2AF	U2 small nuclear RNA auxiliary factor
